# Detection of congenital generalized lipodystrophy mutations by next-generation sequencing: time for a new approach

**DOI:** 10.1186/1758-5996-7-S1-A260

**Published:** 2015-11-11

**Authors:** Aline Dantas Costa Riquetto, Lucas Santos de Santana, Lílian Araújo Caetano, Antônio Marcondes Lerário, Joya Emilie Menezes Correia-Deur, Márcia Nery, Alexander Augusto de Lima Jorge, Milena Gurgel Teles

**Affiliations:** 1 HCFMUSP, São Paulo, Brazil

## Background

Congenital generalized lipodystrophies (CGL) or Berardinelli-Seip Congenital Lipodystrophy (BSCL) are rare autosomal recessive disorders with reduction of subcutaneous and visceral adipose tissue, associated with deregulation of lipidic and glycidic metabolism, most of them developing insulin resistance and diabetes mellitus during the second decade of life. There are four CGL syndromes described (CGL-1 to 4) caused by mutations in AGPAT2, BSCL2, CAV1 and PTRF. The mutations in AGPAT2 and BSCL2 are responsible for 95% of reported cases worldwide and 87% in Brazilian reported cases. BSCL2 variants usually lead to more severe symptoms in comparison to AGPAT2 ones.

## Objective

To report the genotype from 5 different CGL patients using next-generation sequencing (NGS) approach.

## Materials and methods

We have studied 5 unrelated individuals with CGL. Physical evaluation and biochemical measurements were performed in all patients. CGL genes were analyzed using NGS with complete sequence coverage of the coding regions and splice junctions. We have used a diabetes monogenic panel that included familial partial lipodystrophy genes and the four CGL genes (AGPAT2, BSCL2, CAV1, PTRF).

## Results

Genetic analysis identified disease-causing previously described variants in all 5 studied patients: 3 in gene AGPAT2 and 2 in BSCL2. The three AGPAT2 variants found were c.646A>T/p.K216*; c.493-2A>G/IVS4 and g.15373_16409del1037/c.366_492+910del1037 and the two BSCL2 were c.412C>T/p.R138* and c.193_193delinsGGA. All patients had generalized fat loss in first year of life; 100% had hypertriglyceridemia and 80% (4/5) precocious diabetes mellitus, 3 of them with high insulin doses intake. Four in five patients (80%) had hepatomegaly and 60% (3/5) had hepatic steatosis. Both patients with BSCL2 mutations had cardiac complications.

## Conclusions

As clinical phenotype varies among molecularly distinct forms of CGL, understanding molecular defects is helpful in genetic counseling and prenatal diagnosis of affected families and in improving specific therapeutic interventions. We have developed a NGS panel including all known genes causing CGL so far. A NGS approach allows saving time and possibly cost in identifying the etiology of CGL, leading to a better follow-up of each patient.

